# Omega-3 Fatty Acid Based Nanolipid Formulation of Atorvastatin for Treating Hyperlipidemia

**DOI:** 10.15171/apb.2019.031

**Published:** 2019-06-01

**Authors:** Revathy Sreedhar, Vrinda Sasi Kumar, Anil Kumar Bhaskaran Pillai, Sabitha Mangalathillam

**Affiliations:** ^1^Department of Pharmaceutics, Amrita School of Pharmacy, Amrita Vishwa Vidyapeetham, Kochi, Kerala, India.; ^2^Department of Pharmacology, Amrita School of Pharmacy, Amrita Vishwa Vidyapeetham, Kochi, Kerala, India.

**Keywords:** Hyperlipidemia, Nanolipid carrier, Atorvastatin, Omega-3 fatty acid

## Abstract

***Purpose:*** In the current study, attempts have been made to formulate an omega-3 fatty acid based nanostructured lipid carriers of atorvastatin (AT), for treating hyperlipidemia; and to evaluate their antihyperlipidemic activity using in vitro and in vivo studies.

***Methods:*** Omega-3 fatty acid based AT-loaded nanolipid carriers (NLC) were formulated by the melt emulsification ultrasonication technology. The prepared NLC consist of stearic acid (as solid lipid), omega-3 fatty acid (as liquid lipid), Tween 80, poloxamer 188 (surfactants) and soya-lecithin (co-surfactant).

***Results:*** AT loaded NLCs have a particle size of 74.76 ± 4.266 nm, a zeta potential value of -36.03 ± 1.504 mV and a high drug entrapment efficiency (EE) of 86.70 % ± 0.155. The release of AT from NLCs exhibited a sustained behaviour, which made it an ideal vehicle for drug delivery. MTT assay results indicated that NLCs are compatible with L929 (mouse fibroblast) cell lines. Anti-hyperlipidemic study showed a significant reduction in LDL and TG levels in serum with the orally administered Omega-3 fatty acid based AT loaded NLCs when compared to marketed formulation.

***Conclusion:*** The results demonstrated that the omega-3 fatty acid based NLC has the potential to be a promising nanomedicine for the treatment of hyperlipidemia.

## Introduction

Hyperlipidemia has afflicted humankind since middle ages.^1,2^ Hyperlipidemia is one of the major cardiovascular risk factors. It is a state of decreased levels of high density lipoprotein (HDL) cholesterol (<40 mg/ dL), increased levels of low density lipoprotein (LDL) cholesterol (>190 mg/ dL) and increased triglycerides (TG) in plasma (>200 mg/dL), which ultimately result in the progression of atherosclerosis.^3,4^

Basically the treatment involves two approaches, the non-pharmacological therapy and pharmacological therapy. Pharmacological therapy involves mainly the usage of drugs. Generally, the drugs involved in the treatment of hyperlipidemia are classified as follows; 3- hydroxy-3-methylglutaryl coenzyme A (HMG-CoA) reductase inhibitors (statins), bile acid sequestrants (Resins), Activated lipoprotein lipase (fibric acid derivatives), drugs that inhibit lipolysis and triglyceride synthesis like Nicotinic acid and Omega 3 fatty acids. Among these classes of drugs, the most commonly used one is HMG-CoA reductase inhibitors (statins). Among statins, AT is the commonly prescribed drug in treating hyperlipidemia and coronary artery diseases.^5^ It acts by inhibiting the HMG-CoA reductase enzyme involved in the mevalonate pathway which is responsible for the biosynthesis of cholesterol, thereby reducing the cholesterol level in the liver cells This results in an upregulation of LDL receptors and thereby enhancing the clearance of LDL from plasma.^6^ As per the Biopharmaceutical Classification System (BCS), AT belongs to class II drug.^7,8^ The log P value of 4.2 indicates that the drug is highly lipophilic in nature. Due to high lipophilicity, pre-systemic clearance in gastrointestinal mucosa and P-gp efflux mechanism; the oral bioavailability of AT is only 12%.^9,10^ Therefore, the current work is aimed to formulate a nanolipid carrier (NLC) of AT for oral delivery, which will improve its bioavailability.^11^

NLCs have the ability to form micelles in the GI that are capable of reaching the brush border of absorptive cells of the intestine (enterocytes) via crossing aqueous mucin layer.^12^ Statins have only modest triglyceride lowering effect.^13^ Hence an adjunct therapy is usually recommended. Omega-3 fatty acids block the production of very-low-density lipoprotein (VLDL) cholesterol and TG in the liver, thereby reducing the plasma triglyceride levels in persons with hypertriglyceridemia.^14^ Literatures on human studies showed that consumption of 4 g/day of omega-3 fatty acids increase HDL levels by 1%-3%, reduced serum LDL levels by 5-10%, serum TG levels by 25 -30% and total cholesterol level was not knowingly affected.^15^ So in the present study, the potentially active omega-3 fatty acid was selected as a therapeutically active lipid carrier of AT for formulating NLCs.

Lipid-based drug delivery systems have the ability to improve both the solubility and oral bioavailability of poorly aqueous soluble drugs and thus they have bright future as oral carriers.^16^ Compared to Solid Lipid Nanocarriers, NLCs are the latest version of lipid based nanoparticles with increased loading efficiency and stability.^17^ NLCs possess unordered and imperfect lipid arrangements in their matrix that offers sufficient voids for loading drug molecules. NLCs can be prepared by melt emulsification ultrasonication technology. It possess tremendous advantages like improved oral bioavailability, low systemic side effects, non-existent enzymatic degradation of drugs, free of organic solvents and controlled release characteristics. Hence NLC has wide applications in oral, intravenous, pulmonary and transdermal administration.^18^ The NLC matrix is lipophilic in nature and is useful for the oral administration of lipophilic drugs *via* intestinal lymphatic transport system.^19^ It is reported that, the solid lipid like stearic acid and liquid lipid like omega-3 fatty acid possess good hypocholestrolemic effect.^20,21^ So the use of stearic acid and omega-3 fatty acid as ingredients in AT loaded NLC will be beneficial for the treatment of hyperlipidemia.

## Materials and Methods

AT was a donated product from Caplin point laboratories Ltd. (Pondicherry, India), Omega-3 fatty acid capsules (Triomega) was procured from Sanofi India Ltd (Mumbai, India), Stearic acid & methanol from Sanofi India Ltd. (Mumbai, India), Poloxamer 188 from Research Lab Fine Chem Industries (Mumbai, India), Lecithin soya from Himedia laboratories Pvt. Ltd (Mumbai, India) and Tween 80 from Loba Chemie Pvt. Ltd. (Mumbai, India).

### Preparation of AT loaded NLC

**Figure 1:**
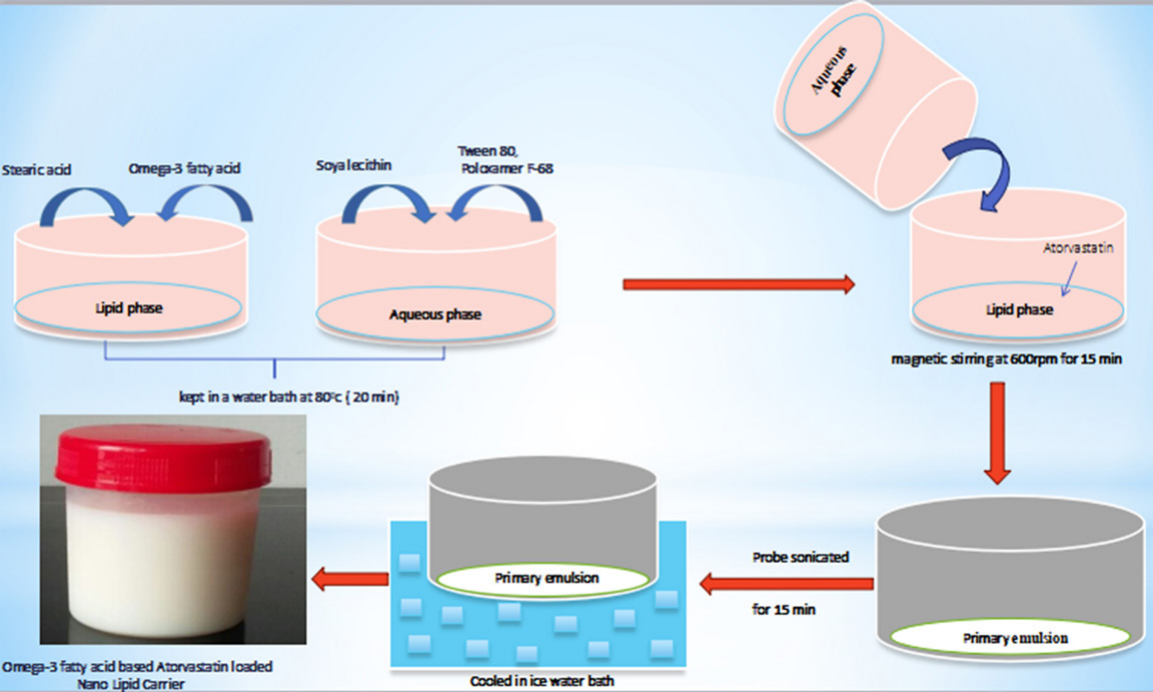
Preparation of Omega-3 fatty acid based AT loaded NLC‏.

The Omega-3 fatty acid based AT loaded NLCs were prepared by melt emulsification followed by Ultrasonication method. In this method, the lipid phase consisting of solid lipid (Stearic acid) and liquid lipid (Omega- 3 fatty acid) were blended and melted above 70°C to form an organic phase. To this lipid phase, AT (10 mg w/w) was added with continuous stirring till homogenous mixture was obtained. Meanwhile, the aqueous phase was prepared consisting of emulsifying agent (Tween 80% w/w), surfactant (Poloxamer 188) and co-surfactant (soya lecithin) dispersed in distilled water. The aqueous phase was added drop wise to lipid phase containing AT at above 70°C with continuous agitation at 600 rpm for 15 minutes to get a primary emulsion. The pre-emulsion was then sonicated (using probe sonicator) for 15 minutes at a frequency of 50 Hz to obtain Omega-3 fatty acid based AT loaded NLC. Subsequently the dispersion was cooled in ice water bath for quick congealing. The prepared dispersion of NLC was stored in air tight container at 4°C.^22,23^ The schematic representation of the preparation of Omega-3 fatty acid based AT loaded NLC is given in Figure 1.

### Determination of entrapment efficiency

In this study, indirect method was used to find out the EE of the prepared formulation. NLC formulation of 5 mL was diluted with 20 mL of distilled water and was centrifuged at 15 000 rpm for 30 minutes. The supernatant was analysed spectrophotometrically at 245 nm. The EE was determined using the formula^24^

$$\%EF=\frac{\left(w_{i}-w_{f}\right)}{w_{i}}\times 100$$

W_i_ : Amount of initial added drug

W_f_ : Amount of drug present in the supernatant after centrifugation of the formulation

### Characterization of AT loaded NLC

The size distribution and surface charge (measurements of zeta potential) of NLC systems were studied by dynamic light scattering (DLS). Scanning electron microscopy (SEM) was used to reconfirm the average size and surface morphology of the NLC systems. Further characetrization was done by Fourier transform infrared (FTIR), X-ray diffraction (XRD) and Differential scanning calorimetry (DSC) analysis.

### FTIR spectroscopy

FTIR was used to assess the drug excipient interaction. Samples were prepared by KBr disc preparation method (Bomem MB-100, Quebec, Canada). Then the powder was added in the cell of FTIR instrument. The FTIR spectra in the range of 4000 - 400 cm^-1^ was recorded.^25^

### Determination of particle size and surface charge

The particle size and surface charge of the plain and AT loaded NLCs were determined using DLS-ZP/Particle sizer Nicomp TM 380 ZLS (California, USA). The particle size was determined as the mean hydrodynamic diameter (*z-average*) and zeta potential value gives an indication of the physical stability in case of colloidal systems. Readings were performed at 25°C. Samples were diluted by double distilled water prior to analysis.^26,27^

### Determination of surface morphology

Morphological characteristics of particles were observed by JEOL JSM-6490 LA variable pressure SEM (Tokyo, Japan). The diluted NLCs were sonicated and few drops were kept on the grid and dried. The image was taken at 25 ± 2°C.^28^

### DSC analysis

DSC analysis was done for active drug AT, plain NLC and Omega-3 fatty acid based AT loaded NLC using DSC-60 (Shimadzu, Japan). DSC aluminium cells and blank DSC aluminium cell were used as sample holder and reference respectively. 2-3 mg sample was used for thermogram analysis and was recorded in the range of 35-300°C at a heating rate of 10°C/ min under atmospheric nitrogen.^29^

### XRD

XRD data allows differentiation between crystalline and amorphous material since it may affect the rate of dissolution and bioavailability of the drug. By using a high-power powder X-ray diffractometer, Malvern Panalytical (Almelo, Netherlands) the XRD spectra of drug and NLCs were taken. Copper was used as target. The products were examined at a 2θ angle range of 2°-45°.^30^

### In vitro drug release study of AT loaded NLC

Dialysis membrane method was used to perform the *in-vitro* drug release study of Omega- 3 fatty acid based NLC loaded with AT. 4 ml of freshly prepared AT loaded NLC was placed in a dialysis bag and was dipped in 30 mL dissolution medium (PBS pH 6.8) maintained at 37 ± 0.5°C at 50 rpm. At different time points, 5 mL of sample was withdrawn from the dissolution medium and to maintain the sink condition the same amount of media was replaced. By using UV-visible spectrophotometer, the withdrawn samples were analyzed for the drug content at 245 nm and the experiment was done in triplicate.^31-34^

Type of kinetic release was determined by quantification of R^2^ value for each kind of kinetic models like zero order, first order, Higuchi, Korsmeyer-Peppas model corresponding to the data obtained during in vitro drug release study.^35^

### Ex vivo skin permeation study

*Ex-vivo* permeation study was carried out using rat small intestine in order to predict intestinal drug permeation from NLC. The isolated intestine was washed with PBS pH 7.4. Intestine was filled with 1 mL of NLCs and tied at both ends. This NLC filled intestine was placed in an organ bath with constant aeration and temperature maintained at 37°C. 35 ml of PBS was taken in receiver compartment. 3 mL of sample at different time interval (15, 30, 45, 60, 90, 120 minutes) was withdrawn and replaced with same volume of fresh buffer. The drug concentration was measured by UV visible double beam spectrophotometer, Malvern Panalytical (Almelo, Netherlands) at 245 nm. Buffer solution is considered as blank.^36^

### In vitro hemolysis assay

Fresh human blood was used to evaluate the hemocompatibility of formulations by *in vitro* hemolysis assay. Collected blood samples in a vacutainer containing Acid Citrate Dextrose (ACD) as coagulant. To 1ml of blood sample, 100 µL of different concentrations (20, 40, 60, and 80 µg/mL) of NLCs were added and incubated at 4°C for 2 hours. Plasma was separated by centrifugation for 10 min at 4500 rpm. Separated plasma of 100 µL was mixed with 1 µL of 0.01% Na_2_CO_3_. The OD values were read at 450, 380 and 415 nm using the equation given below. The plasma hemoglobin can be calculated using the following equation.

Plasma Hb= {(2A415)-[A380 + A450] - 76.25}

The obtained values were compared with positive control (0.1% triton) and negative control (normal saline). Triplicate readings were taken.^37^

### In vitro cytocompatibility study

*In vitro* cytocompatibility of the Omega-3 fatty acid based AT loaded NLC was tested on L929 cells (Mouse dermal fibroblasts). MTT [3-(4,5-dimethylthiazole-2-yl)-2,5-diphenyl tetrazolium] assay was used to assess the cytocompatibility of the optimized formulations. For the assay, different concentrations of the samples were prepared by dilution of stock solution/formulations using Dulbecco’s Modified Eagles Medium (DMEM). The counted cells were seeded in 96 well plates at a density of 10 000 cells/cm^2^. After reaching confluency, the cells were treated with the samples for specified period of time. Triton treated cells served as negative control and untreated cells in media acted as positive control. After specified time period, the cells were treated with MTT and kept again for 4 hours in CO_2_ incubator for formazan crystal formation. Finally the formed crystals were dissolved using solubilization buffer and the optical density was measured at 570 nm using Elisa plate reader, Bioline technologies (Thane, India).^38^

### Stability studies

The NLC dispersions were filled in glass wares and stored at three different conditions at 30 ± 2°C (room temperature), at 4 ± 2°C (refrigerator condition), and at 40°/ 75% RH (accelerated condition) for a period of three months. The physical stability of the formulation was examined visually for appearance, colour and odour in every 30 days. The change in particle size and EE were measured and recorded.^38^

### In vivo studies

For*in vivo* studies, male albino Wistar rat (weight: 150-200 g) were procured from Central lab animal facility, AIMS, Kochi under Institutional Animal Ethics Committee (IAEC) approval with reference no: IAEC/ 2017/ 3/ 6. They were grouped and housed in wire mesh cage one week prior to the study for acclimatizing them with laboratory conditions (temperature at 22 ± 2℃ and RH at 50 ± 15%). Animals were handled as per the guidelines of the Committee for the Purpose of Control and Supervision of Experiments on Animals (CPCSEA). CPCSEA is a statutory body formed by the Act of the Indian Parliament under the Prevention of Cruelty to Animals Act 1960.^39^

### Pharmacodynamic studies

The anti-hyperlipidemic effects of AT formulation were compared with marketed AT, Omega-3 fatty acid and placebo formulation using poloxamer i.p injected model. Male Albino Wistar rats of weight between 150-200 g were used for determining the antihyperlipidemic effect of NLC formulation. A single intraperitoneal dose of poloxamer 407 is known to cause a rapid onset of hyperlipidemia.^40^ Poloxamer 407 increases serum lipoproteins via its actions at various levels in lipid metabolism, largely by inhibiting lipoprotein lipase, which facilitates the hydrolysis of TG. Poloxamer 407 also causes indirect stimulation of HMG-CoA reductase which is involved in cholesterol biosynthesis.^39^ For inducing hyperlipidemia in rats, a colloidal solution of poloxamer 407 was prepared by dissolving 7 g in 52 mL of cold normal saline (0.13 g/mL), which was stored at 4°C. Rats were anesthetized with isoflurane and oxygen and the poloxamer 407 solution was carefully injected intraperitoneally at a dose of 0.5 to 1 g/kg.

The Wistar rats were divided into six groups. Each group containing four animals were administered with different formulations. Group I served as negative control, group II was treated with plain NLC, group III with optimized AT-NLCs (dose 1) formulation, group IV with optimized AT-NLCs (dose 2), group V treated with marketed formulation of omega 3 fatty acid and group VI treated with marketed formulation of AT. After induction of hyperlipidemia at the 12th hour, drug and formulations were orally administered to animals. This procedure was repeated for 7 days. A blood volume of 0.4 mL was withdrawn from the retro orbital plexus on each day upto 7 days of induction of hyperlipidemia. Using *in-vitro* diagnostic kit, the separated serum was analyzed for HDLs, LDLs, TC, and TGs.^41,42^

### Statistical analysis

All data was expressed in mean ± standard deviation after triplicate sample analysis. Statistical significance was assessed by paired student’s two tailed *t* test, wherever necessary. A value of *P* < 0.05 is considered to be statistically significant.

## Results and Discussion

AT loaded NLC was formulated by melt emulsification- ultrasonication technique. Poloxamer 188 and soya lecithin were used as surfactants, respectively. The method and composition of the NLCs were optimized, and they were further considered in terms of the size, ζ-potential, shape, EE%, in vitro release and stability. Table 1 shows the major properties of the formulated AT loaded NLC in this study.

**Table 1:** Major ingredients and specifications of the optimized formulation of AT loaded NLC

**Ingredients of optimized atorvastatin loaded nanolipid carrier**	**Particle size (nm)**	**Surface charge (mV)**	**Entrapment efficiency (%)**
**Aqeous Phase**	87.29 ± 6.68	-36.03 ± 1.50	86.70 % ± 0.15
Tween 80			
Poloxamer 188 (surfactant)			
Soya lecithin (co- surfactant)			
**Lipophilic phase**			
Stearic acid			
Omega-3 fatty acid			
AT			

### Preparation of drug loaded NLC

The AT loaded NLCs were prepared using varying concentrations of solid and liquid lipid by the melt emulsification- ultrasonication technique as shown in Figure 1. It is reported that the formulations prepared with surfactant mixtures such as poloxamer 188 and soya phospholipid have lesser particle size and greater stability on storage compared to lipid nanoparticles stabilized with one surfactant alone. The combination of lipophilic and hydrophilic surfactants such as soya lecithin and poloxamer 188 in 1:1 molar ratio is known to be helpful in improving the stability of the system hence in the development of AT-NLCs the same combination was selected.^40^ Tween 80 was selected as the surfactant. The major ingredients and specifications of the optimized AT loaded NLCs are shown in Table 1.

### Entrapment efficiency results

The prepared formulation showed an average % EE of 86.70 % ± 0.15. The addition of liquid lipids in to solid lipids leads to a great disordered disturbance and imperfections in the crystal lattice that provides high space to accommodate drug molecules, thus resulting in high EE. The EE mainly depends on the nature of the drug and lipids as well as lipophilic nature of the drug with a log P of 6.6 may be another reason for the high EE.

### Characterization of atorvastatin loaded NLC

#### FTIR studies

**Figure 2:**
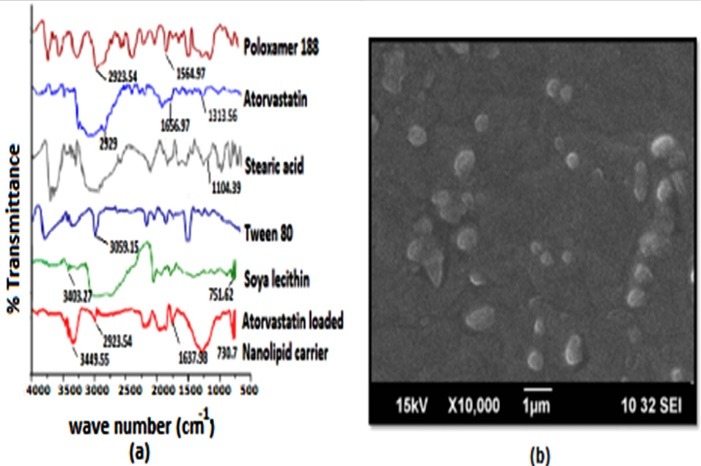
(a) FTIR spectrum of AT, excipients and AT loaded NLC; (b) SEM image of AT loaded NLC.

The FTIR study of AT loaded NLC was done to study the potential chemical interaction between the drug and the excipients, which is showed in Figure 2a. The FTIR spectrum of pure AT, stearic acid, poloxamer 188, soya lecithin, and tween 80 showed characteristic peaks at 2955.15 cm^-1^ (CH stretching), 1313.56 cm^-1^ (CN stretching), 3059.15 cm^-1^ (C-HO-stretching alcoholic group), 1564.97 cm^-1^ (C=O-stretching amide group), 3403.27 cm^-1^ (NH stretching), 1656.97 cm^-1^ (C=C bending), 751.62 cm^-1^, 696.95 cm^-1^ (CF stretching), 1104.39 cm^-1^ (OH bending), 2929 cm^-1^ ( aliphatic CH stretch), which was already reported by our group in many past studies. However, spectra of drug loaded NLC showed shifts corresponding to alcoholic OH stretching from 3059.15 cm^-1^ to 3449.55 cm^-1^, a peak at 2923.54 cm^-1^ assigned to aliphatic C-H stretching , peaks at 1637.98 cm^-1^ and 730.70 cm^-1^ indicating C=O–stretching amide group and C-F- stretching. All the significant peaks in AT and other excipients were retained in the final formulation. Hence it is confirmed that the drug is encapsulated inside the lipid structure without any significant chemical interaction between drug and the excipients.

### Particle size and surface charge results

It is reported that application of two surfactants (poloxamer 188 and soya lecithin) instead of only one type of surfactant, this would result in small and stable NPs.^34^ The size of both AT loaded NLC and plain NLC were between 70 and 85 nm. The average particle size of AT loaded NLC was found to be 87.29 ± 6.68 nm. The incorporation of higher level of liquid lipid and optimum concentration of hydrophilic surfactant poloxamer 188 caused a reduction in particle size. The obtained zeta potential value was found to be -36.03 ± 1.50 mV. Zeta potential values greater than -30/ +30 mV demonstrate the stability of the colloidal suspension. The concentration of surfactant present in NLC is responsible for the negative zeta potential value.^43^ Based on the ingredients used in the formulations, the achieved zeta potential may be associated with the free electrons which are present in different atoms.^34^

### SEM results

To study the morphological characteristics of the particle SEM analysis was also done. The SEM image of AT loaded NLC is shown in Figure 2b. The SEM images revealed that the particles are almost spherical in shape and uniformly dispersed without any aggregation.

### DSC results

**Figure 3:**
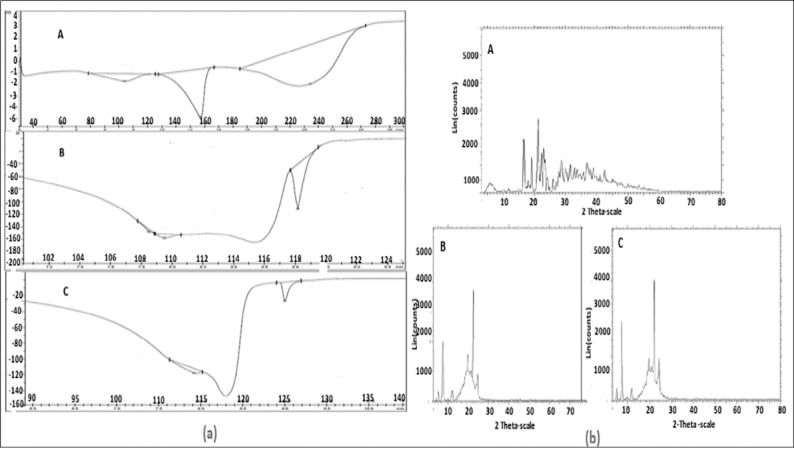
DSC thermogram of A) Pure AT B) Plain NLC C) AT loaded NLC, b) XRD diffractogram of A) Pure AT B) Plain NLC C) AT loaded NLC.

From the DSC data, a sharp endothermic peak at 156.95°C as shown in Figure 3a was observed which corresponds to the melting point of crystalline pure drug. In the DSC thermogram of AT loaded NLC, appearance of endothermic peak equivalent to melting point of pure AT was absent and the absence of this peak in the DSC thermogram is usually the sign of dissolved, amorphous and molecularly dispersed drug within the lipid or the drug-lipid interaction (plasticizing effect of the AT) or a polymorphic change of the drug which can be detected as peak shifts in the DSC thermogram. Therefore, it could be concluded that AT in the NLCs is either in an amorphous form, in molecular dispersion or a solid solution state in the lipid matrix as there is complete disappearance of the endothermic peak.^44^

### XRD results

XRD diffractograms of AT, Plain NLC and AT loaded NLC are presented in Figure 3 b. The XRD patterns of AT showed numerous diffraction peaks at diffraction angles (2θ) 21.14°, 22.18°, 22..83°, 24.02°, 16.60°, 18.98°, 28.69°, 36.75°, 42.58° and 25.84°, confirming its crystalline pattern. In case of plain and AT loaded NLC, these diffraction peaks have disappeared demonstrating the existence of drug in the amorphous form, confirming the DSC data.^45^

### In vitro drug release studies results

The *in vitro* drug release of drugs from NLC is depend on nature of drug which is incorporated. In case of lipophilic drugs, the drug is concentrated mostly near the surface of the particle. So the drug release will be fast and there may be burst drug release after the administration of the formulation. When the drug concentration is higher or near to its solubility limit in lipid phase, the drug is located in the core of the carrier model thereby providing a sustained or prolonged drug release.^34^

**Figure 4:**
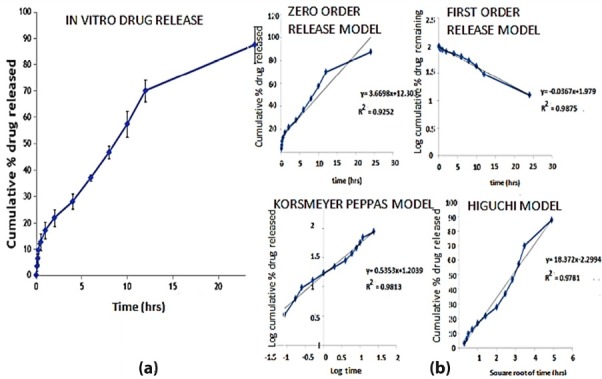
a) In vitro drug release of AT from optimized NLC, b) Kinetic modeling of in vitro drug release.

The *in vitro* release of AT from formulated NLC was investigated by dialysis membrane method in PBS (pH 6.8) at 37 ± 0.5°C. Figure 4a shows the mean cumulative release profile of AT from the formulated NLC. The release of AT from the NLC shows a biphasic release pattern i.e. initially there is a burst release followed by a sustained release. In the first 2 hours about 21% of AT was released which may due to the drug release from the surfactant layer of NLC and this indicates a release behaviour similar to that of conventional dosage form in the GIT. After the initial burst release it showed a sustained release and about 87.51 % was released within 24 hours. This slow release would be advantageous considering the possible conversion of NLCs to micelles in the GIT and the enhanced intestinal absorption expected from this. So we can assume that most of the remaining drug release would happen in the systemic circulation once the drugs in micelle are absorbed through chylomicron mediated pathway.

### Kinetic modeling of AT loaded NLC

The kinetic release models for the AT loaded NLC was performed and the coefficient of determination (R^2^) for each model was determined based on the data obtained during *in vitro* release study (Table 2). Based on our findings, the release data (i.e., AT release from NLC for 24 hours) were kinetically best fitted with the first order kinetic model (R2= 0.987)i.e., drug release is concentration dependent.^35^

**Table 2:** Kinetic data analysis of optimized AT loaded NLC

**Formulation**	**R** ^ 2 ^				
	**Zero order**	**First order**	**Higuchi model**	**Korsmeyer-Peppas model**	**n**
Optimized NLC	0.9252	0.9875	0.9781	0.9813	0.53

### Ex vivo skin permeation study

**Figure 5:**
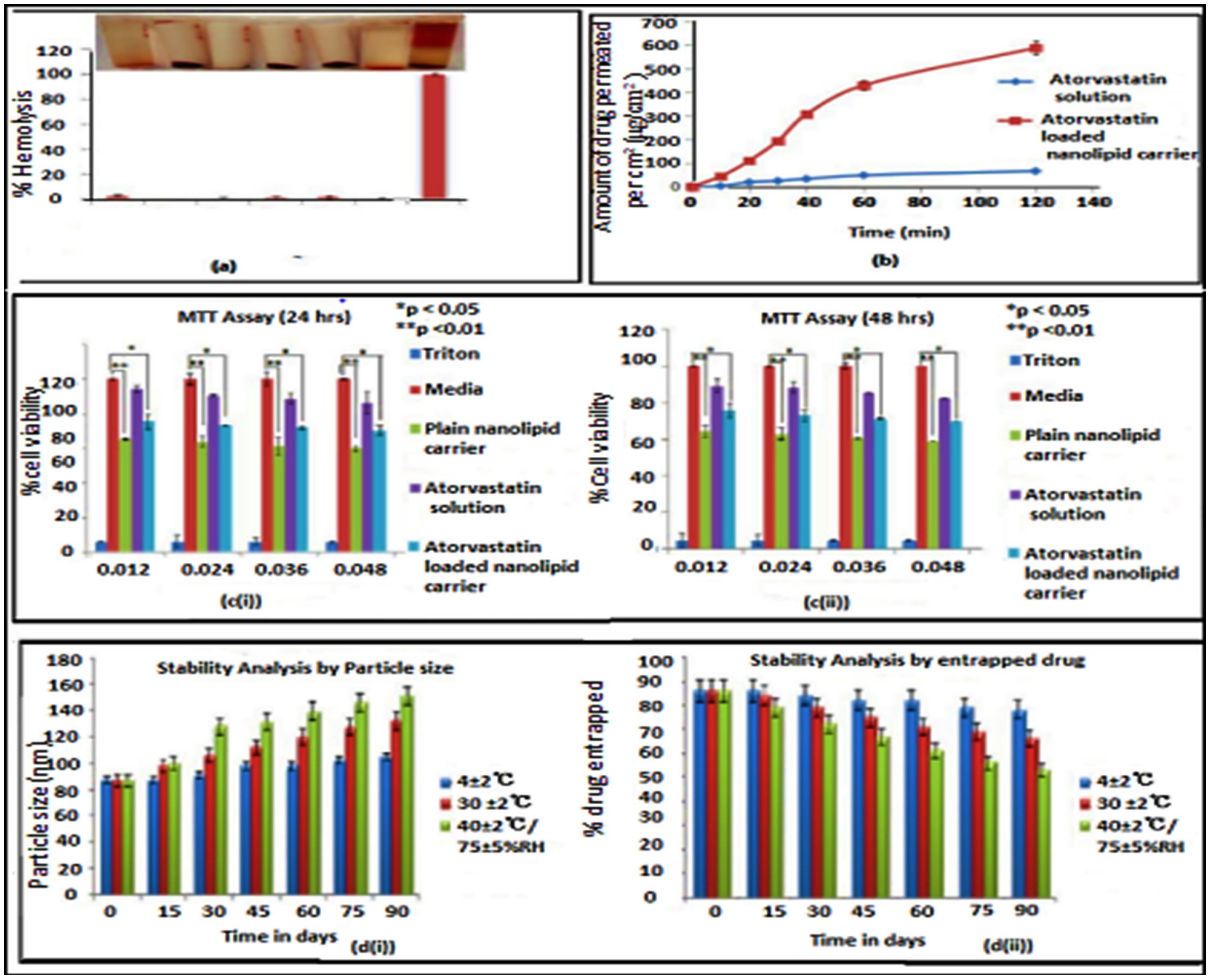
a) Hemolysis assay results of NLCs b) Ex vivo permeation result of AT loaded NLC c) Cytocompatibility studies (24 hours i) and 48 hours ii)) of AT loaded NLC in L929 cells; A) Triton B) Media C) Plain NLC D) AT loaded NLC d) Stability studies of AT loaded NLC in different temperatures in 3 months; d i) based on particle size analysis d ii) based on drug entrapment.

The permeation study using rat intestine was performed to study the intestinal permeation of AT loaded NLC. The cumulative amount of drug permeated across rat ileum is plotted against time as shown in Figure 5b. It was found that 587.15 ± 0.41 µg of AT was permeated from NLC per cm^2^ area of ileum when compared to plain drug solution having permeation of 70 ± 0.26 µg/cm^2^. Lipid based nanoparticles have a major role in increasing drug transport from intestine because they increase intestinal permeability and the surfactants which are present in the formulation mitigate intestinal efflux by inhibition of the P-glycoprotein efflux pump which is present in the villus tip of enterocytes in the gastrointestinal tract. Digestive lipids comprising of dietary lipids such as fatty acids, glycosides, phospholipids, cholesterol esters as well as various synthetic derivatives increase the transport of the drug from the intestine.^44^

### In vitro hemolysis assay results

Hemolysis assay was performed to check the compatibility of the AT-NLC with blood, since it may reach systemic circulation on oral administration. The degree of hemolysis is a sensitive indicator of the extent of damage to RBC. Figure 5a shows the results of hemocompatibility studies of NLCs. In the present study, the different concentrations of optimized formulations were tested and showed no evidence of hemolysis (<0.5%) after incubation for specified time period. The obtained results were compared with standard normal saline and the positive control Triton X. As per the results obtained the hemolytic ratios of the NLCs were below 5 %, which is the normal percentage limit of hemolysis for biomaterials in accordance with ISO/TR 7406. It confirms that the formulation is compatible with blood components.

### In vitro cytocompatibility study results

*In vitro* cytocompatibility screening was performed using L929 cell line by MTT assay. Prepared NLC formulation was tested for cytotoxicity at different concentrations like 0.012, 0.024, 0.036, and 0.048 mg/mL. Percentage viability was found to be 89.32 to 69.21 (minimum to maximum dose) which indicates that prepared NLC formulation is not toxic towards the normal cell line. The % viability of L929 Cells treated with prepared NLC after 24 and 48 hours is shown in Figure 5ci and cii.

### Stability studies

The stability study of AT loaded NLC was carried out at different temperatures for three months which is shown in Figure 5di and dii. The particle size and percentage drug entrapment were determined after storing the formulation at three different storage conditions such as 4 ± 2°C (refrigeration condition), 30 ± 2°C and 40 ± 2°C, 75 ± 5% RH (accelerated condition). At refrigerated temperature, there was only a slight change in particle size from 87.29 ± 2.68 nm to 90.54 ± 0.25 nm and drug entrapment from 86.70 % ± 0.15 to 79.3 % ± 0.23. When the formulation was stored at room temperature, the particle size and EE was found to be 130 ± 0.9 nm and 65 % ± 1.0 respectively. The particle size desirable for the transport across the intestine after oral administration is 300 nm, thus an increase in particle size seen with storage under room temperature will not affect the therapeutic benefit of the formulation. Upon storage at accelerated temperature, the formulation showed an increase in the particle size (150 ± 0.2 nm) and decrease in entrapped drug (55% ± 0.5), this might be due to leaching of drug from the NLC structure. These results suggest that the optimum storage condition for AT loaded NLC is refrigerated condition since there was no significant change in the particle size and percentage entrapment of the stored formulation.

### In vivo studies of AT loaded NLC

#### Pharmacodynamic evaluation

**Figure 6:**
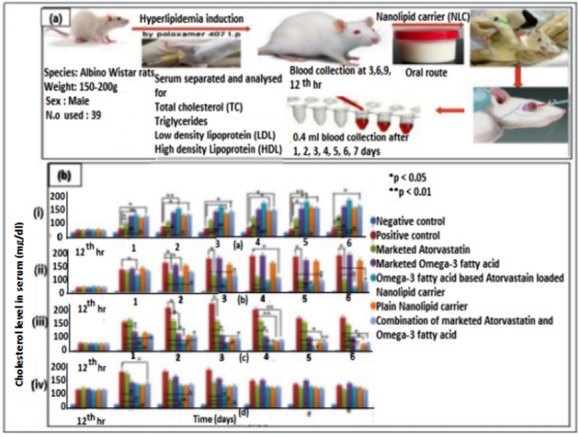
a) Strategy of *in vivo* study i) HDL level, ii) LDL level, iii) Triglyceride level, iv) Total cholesterol level after treatment with different formulations b) cholesterol level in serum after treatment with different formulations.

As expected, i.p injection of poloxamer 407 led to elevation of LDL, triglyceride, total cholesterol and slight decrease in HDL values, which were retained over the period of study for 7 days in hyperlipidemic control group (positive control). The positive control groups were treated with marketed AT, marketed omega- 3 fatty acid, combination of marketed formulations of AT and Omega-3 fatty acid, plain NLC and Omega-3 fatty acid based AT loaded NLC. On the first day itself there was a significant elevation in serum lipids and lipoproteins in poloxamer 407 induced positive control groups when compared with negative control. A decreased level of LDL was observed in Omega-3 fatty acid based AT loaded NLC and showed an improved HDL level (150 mg/dL) compared to the marketed AT. Marketed omega-3 fatty acid, combination of AT and Omega-3 fatty acid and plain NLC also showed an improved HDL level. It is reported that Omega-3 fatty acids decreases hepatic VLDL-TG production and secretion by decreasing enzymatic conversion of acetyl CoA to fatty acids.^45^ The LDL level and TG level was significantly reduced after the treatment with Omega-3 fatty acid based AT loaded NLC compared to the marketed AT and the combination of AT and omega-3 fatty acid. But there was no significant reduction in the LDL and TG level among Marketed omega-3 fatty acid and plain NLC treated groups. Except the marketed omega-3 fatty acid, all other formulation showed a slight reduction in total cholesterol level but not significant with respect to the positive control. So from*in vivo* studies it was found that the hypocholesterolemic effect of Omega-3 fatty acid and AT is more in combination compared to its individual forms. The elevated HDL level and significant decrease in LDL and TG levels by Omega-3 fatty acid based AT loaded NLC reflects the superiority of the NLC formulations by lowering the hyperlipidemic condition. Strategy of *in vivo* study and its results are shown in Figure 6a and 6b.

## Conclusion

In the present study, Omega-3 fatty acid based AT loaded NLC was investigated. The pharmacodynamic study proved the profound hypolipidemic effects of Omega-3 fatty acid in combination with AT which is better than their individual forms. The reported advantage of NLC of improving the bioavailability of loaded drug was leveraged for preparing Omega-3 fatty acid based AT loaded NLC. Serum lipid profiles demonstrated that Omega-3 fatty acid based AT loaded NLC has significant lipid lowering potential compared to the marketed formulation. The hypocholesterolemic functions of Omega-3 fatty acids are probably due to inhibition of VLDL, cholesterol and TG synthesis in the liver. In conclusion, Omega-3 fatty acid based NLCs of poorly water soluble AT was an effective approach for improving its oral bioavailability and antihyperlipidemic activity.

## Ethical Issues

Not applicable.

## Conflict of Interest

The authors have no conflict of interests.

## Acknowledgments

We are thankful to Caplin Point Laboratories Ltd, Pondicherry for providing gift sample of AT. The great support from all colleagues at Amrita School of Pharmacy and Amrita Centre for Nanosciences is also acknowledged.
